# Neural conditional ablation of the protein tyrosine phosphatase receptor Delta PTPRD impairs gliogenesis in the developing mouse brain cortex

**DOI:** 10.3389/fcell.2024.1357862

**Published:** 2024-02-29

**Authors:** Francisca Cornejo, Nayhara Franchini, Bastián I. Cortés, Daniela Elgueta, Gonzalo I. Cancino

**Affiliations:** ^1^ Center for Integrative Biology, Faculty of Sciences, Universidad Mayor, Santiago, Chile; ^2^ Department of Cell and Molecular Biology, Faculty of Biological Sciences, Pontificia Universidad Católica de Chile, Santiago, Chile

**Keywords:** gliogenesis, glia, brain cortex, PTPRD, neural precursor cells, astrocytes, oligodendrocytes, neurodevelopment

## Abstract

Neurodevelopmental disorders are characterized by alterations in the development of the cerebral cortex, including aberrant changes in the number and function of neural cells. Although neurogenesis is one of the most studied cellular processes in these pathologies, little evidence is known about glial development. Genetic association studies have identified several genes associated with neurodevelopmental disorders. Indeed, variations in the *PTPRD* gene have been associated with numerous brain disorders, including autism spectrum disorder, restless leg syndrome, and schizophrenia. We previously demonstrated that constitutive loss of *PTPRD* expression induces significant alterations in cortical neurogenesis, promoting an increase in intermediate progenitors and neurons in mice. However, its role in gliogenesis has not been evaluated. To assess this, we developed a conditional knockout mouse model lacking *PTPRD* expression in telencephalon cells. Here, we found that the lack of PTPRD in the mouse cortex reduces glial precursors, astrocytes, and oligodendrocytes. According to our results, this decrease in gliogenesis resulted from a reduced number of radial glia cells at gliogenesis onset and a lower gliogenic potential in cortical neural precursors due to less activation of the JAK/STAT pathway and reduced expression of gliogenic genes. Our study shows PTPRD as a regulator of the glial/neuronal balance during cortical neurodevelopment and highlights the importance of studying glial development to understand the etiology of neurodevelopmental diseases.

## Introduction

The brain cortex is a stratified structure involved in higher brain functions. During mammalian cortical development, neural precursor cells (NPCs) or radial glial cells are first differentiated into neurons and later, at perinatal stages, switch to a glial fate, differentiating into astrocytes and oligodendrocytes, directly or indirectly through intermediate glial precursors (Lin et al., 2021). In the mouse cortex, gliogenesis starts around embryonic day (E) 16 and peaks at postnatal day (P) 0 (Sauvageot and Stiles, 2002; Miller and Gauthier, 2007). Even though there are differences in the spatial, temporal, and transcriptional features of mammalian neurodevelopment, the cortical NPCs lineage progression and hierarchy are conserved among species (Lin et al., 2021). The human brain has approximately equal proportions of neurons and glial cells. Thus, maintaining this proportion is essential for proper brain organization. During neurodevelopment, glial cells regulate neuron number, axon growth and guidance, synaptogenesis, and synaptic maturation, among other cellular processes (Reemst et al., 2016; Allen and Lyons, 2018). Notably, neurons lacking glial trophic support do not integrate into neural networks and die (Oppenheim et al., 2013). Also, from birth to adolescence, glial and neuron-glia interactions determine most of the brain assembly, defining its final organization. Therefore, neurodevelopmental disorder mechanisms can only be elucidated entirely by considering gliogenesis.

NPCs balance between cellular proliferation and differentiation into neurons and glia is essential for proper brain development and function. Impairments in this balance may induce abnormal brain formation and neurodevelopmental disorders frequently produced by genetic mutations. Among those genes, it has been observed that mutations in the Protein Tyrosine Phosphatase Receptor Delta (PTPRD) are involved in the development of several brain disorders, such as autism spectrum disorder (ASD) (Pinto et al., 2010; Levy et al., 2011; Gai et al., 2012; Liu et al., 2016), attention deficit hyperactivity disorder (ADHD) (Elia et al., 2010), schizophrenia (Li et al., 2018), obsessive-compulsive disorder (OCD) (Gazzellone et al., 2016), anxiety (Li et al., 2023) and restless legs syndrome (Schormair et al., 2008; Yang et al., 2011; Cornejo et al., 2021). Interestingly, we have previously shown that PTPRD constitutive knockout (KO) mice have impaired cortical embryonic neurogenesis with aberrantly increased neurogenic intermediate progenitor cells and neurons and neuronal mislocalization, in part due to hyperactivation of the receptor tyrosine kinase TrkB and PDGFRβ and their downstream signaling pathway MEK-ERK (Tomita et al., 2020). Since neurons and glia are differentiated from the same NPCs pool (Zhang et al., 2020; Li et al., 2021; Lin et al., 2021; Shen et al., 2021), we asked whether *PTPRD* mutations could also induce alterations in glial cell development. Indeed, it has been previously shown that PTPRD KO mice have delayed myelination in the spinal cord at postnatal stages (P4-P7) (Zhu et al., 2015). However, the molecular mechanism implicated and whether this feature is also induced in other central nervous system areas has yet to be explored.

Here, we used a PTPRD conditional KO mouse (PTPRD cKO), lacking its expression in EMX1+ cells, which originates the telencephalon, to characterize NPCs gliogenic features that could be being altered in the cortex of mice lacking *Ptprd* expression. Compared to our previous results using constitutive PTPRD null mice (Tomita et al., 2020), we observed that some aberrant neurogenic features are replicated in this PTPRD cKO mouse model. Also, our results suggest that gliogenesis is impaired, as shown by a reduced number of glial precursors, astrocytes, and oligodendrocytes, as well as reduced myelinization in the PTPRD cKO postnatal brain cortex. As we show here, this might be due to a reduction in the number of radial glial cells that remain after neurogenesis, an increased neurogenic potential in cortical NPCs lacking *PTPRD* expression, and their reduced activation of the gliogenic intracellular signaling JAK-STAT (Bonni et al., 1997; He et al., 2005; Hong and Song, 2014; Sloan and Barres, 2014; Han-Chung et al., 2016; Lee et al., 2016; Takouda et al., 2017). Given the critical role of glial cells in physiological brain function, and since neurodevelopmental disorders often imply alterations in the number and function of glial cells (Tartaglia et al., 2001; Hegedus et al., 2007; Paquin et al., 2009; Zdaniuk et al., 2011), our results highlight the importance of studying brain disorders etiology by addressing the different cell types that could be involved, and not only from a neuronal perspective.

## Materials and methods

### Animals

The C57BL/6N-A < tm1Brd > Ptprd < tm2a (KOMP)Wtsi>/WtsiOrl (PTPRD KO) mice were purchased from Wellcome Trust Sanger Institute. To generate the PTPRD cKO mice, we crossbreed PTPRD KO mice with FLP recombinase mice (B6. Cg-Tg (Pgk1-flpo)10Sykr/J) purchased from Jackson laboratory to eliminate the LacZ and Neo cassettes, and to obtain only the LoxP sites flanking exon 2. Subsequently, the FLP background was removed, leaving only pure LoxP sites. Afterward, they were crossed with Emx1-Cre mice (B6.129S2-*Emx1* ^*tm1(cre)K*^ *rj*/J) to obtain a conditional PTPRD null mouse for telencephalic neural precursor cells (Gorski et al., 2002). Mice were genotyped as described previously (Tomita et al., 2020) and the primers used are the following: Ptprd_111,547_F: 5′-TCA​CCT​CGC​TGT​TCT​TCC​TG-3’; Ptprd_111,547_R: 5′- CTT​CTC​AGT​GCC​CAA​CCC​TC-3’; CAS_R1_Term: 5′-TCG​TGG​TAT​CGT​TAT​GCG​CC-3’. All mice had free access to rodent chow and water in a 12-h dark-light cycle room. All animal studies were approved by the Animal Ethics Committee of Universidad Mayor and Pontificia Universidad Católica de Chile.

### Neurosphere cultures

Neurospheres were obtained following the previously detailed protocol (Tomita et al., 2020). Briefly, cortices from E16.5 PTPRD WT or PTPRD cKO mouse embryos were dissected in ice-cold HBSS and mechanically dissociated into a single-cell suspension using fire-polished glass pipettes. Cell density and viability were determined using trypan blue exclusion. Cells were seeded at clonal density (10 cells/mL) in 10 cm culture in serum-free medium (SFM) which contained 1 part low glucose Dulbecco’s Modified Eagle Medium (DMEM) with 1 part Ham’s F-12 Nutrient Mixture (F12), 0.6% glucose, 0.1125% sodium bicarbonate, 5 mM HEPES, 100 μg/mL L-glutamine, and 1% Pen/Strep, and supplemented with 20 ng/mL EFG, 10 ng/mL FGF2, 2% B27 and 2 mg/mL heparin. Neurospheres were cultured at 37°C in 5% CO2 for 7 days *in vitro* (DIV).

### *In vitro* differentiation assay

Neurospheres were processed as previously described (Dittmann et al., 2022) with some modifications. Briefly, 7 DIV floating neurospheres were mechanically triturated 10 times with P1000 pipettor and spun down at 500 g for 7 min. The supernatant was aspirated, and cells were washed in 1 mL of warm neurosphere processing media (SFM supplemented with 2% B27 and 10 ng/mL FGF). Cells were again spun down at 500 g for 7 min, resuspended in 1 mL neurosphere processing media, and triturated again with a P1000 pipettor a maximum of 5 times. Cells were filtered through a 70 μm cell strainer and seeded as adherent monolayers on 12 mm diameter glass coverslips pre-coated with 40 μg/mL Poly-D-Lysine hydrobromide and 4 μg/mL laminin. Adherent cells were maintained in SFM supplemented with 2% B27, 10 ng/mL FGF2, and 20 ng/mL EFG and incubated at 37°C in 5% CO2 for 7 or 14 DIV. The cell seeding density was 39,500 cells/cm^2^.

### Immunocytochemistry and histological analysis

Tissue immunostaining was performed as described (Cancino et al., 2015). Briefly, brain tissues were washed with TBS (Tris-buffered saline), permeabilized with 0.3% Triton X-100 solution in TBS, and incubated in blocking solution (5% BSA and 0.3% Triton X-100 in TBS) for 1 h at room temperature (RT). Then, brain sections were incubated with primary antibodies in blocking solution overnight at 4°C. After TBS washes, brain sections were incubated with secondary antibodies in blocking solution for 1 h at RT. Finally, after TBS washes, sections were counterstained with Hoechst 33,258 and mounted in Dako Fluorescence Mounting Medium.

Coverslips were washed once with TBS and fixed with ice-cold 4% paraformaldehyde for 30 min for adherent cell immunostaining. Then, cells were washed 3 times with TBS and incubated in blocking solution for 1 h at RT. Primary antibodies were incubated in blocking solution overnight at 4°C. Coverslips were washed in TBS 8 times by immersion and incubated with secondary antibody for 1 h at RT. After the secondary antibody was washed in TBS 8 times by immersion, nuclei were stained with Hoechst 33,258 and washed in TBS 3 times by immersion. Coverslips were mounted using Dako mounting medium.

Digital image acquisition was performed using Las X software (Leica Microsystems) on a Leica DMi8 microscope, and cell quantification was performed in ImageJ (Schneider et al., 2012).

### Western blotting

Neurosphere cultures were lysed in RIPA buffer (50 mM Tris, pH 8, 150 mM NaCl, 1% NP-40, 0.1% SDS, 1 mM EDTA) containing 1 mM PMSF (phenylmethylsulfonyl fluoride), 1 mM sodium vanadate, 20 mM sodium fluoride, 10 mg/mL aprotinin, and 10 mg/mL leupeptin. Fifty micrograms of protein lysate were electrophoresed, and western blots were performed as described previously (Barnabé-Heider and Miller, 2003).

### Antibodies

The primary antibodies used for immunostaining were rabbit anti-Pax6 (1:500, Biolegend # 901301), rat anti-Tbr2 (1:250, Invitrogen #14-4875–82), mouse anti-Satb2 (1:300, Abcam #ab51502), rabbit anti-Tbr1 (1:400, Abcam #ab31940), rabbit anti-Sox9 (1:200, Cell Signaling #82630S), rabbit anti-GFAP (1:1000, Dako #Z0334), mouse anti-Olig2 (1:200, Millipore #MABN50), rat anti-MBP (1:300, Millipore #MAB386), mouse anti-NeuN (1:500, Millipore #MAB377), and rabbit anti-Sox10 (1:200, Cell Signaling #89356S). The secondary antibodies used for immunostaining were Alexa Fluor 555-conjugated donkey anti-mouse, anti-rat, and anti-rabbit IgG (1:1000), and Alexa Fluor 488-conjugated donkey anti-rabbit, and anti-mouse IgG (1:1000). The primary antibodies used for immunoblotting were rabbit anti-phospho-Jak1 (1:1000, Cell Signaling #74129T), rabbit anti-Jak1 (1:1000, Cell Signaling #3344T), rabbit anti-phospho-STAT3 (1:1000, Cell Signaling #9145S), mouse anti-STAT3 (1:1000, Cell Signaling #9139S), rabbit anti-β-actin (1:1000, Cell Signaling #4970S), rabbit anti-phospho-MEK1/2 (1:1000, Cell Signaling #9121S), rabbit anti-MEK1/2 (1:1000, Cell Signaling #9122S), rabbit anti-phospho-ERK1/2 (1:000, Cell Signaling #9101S), mouse anti-ERK1/2 (1:000, Cell Signaling #4696S), and mouse anti-HSP90 (1:000, Santa Cruz Biotechnology #13119). The secondary antibodies used for immunoblotting were HRP-conjugated donkey anti-rabbit and anti-mouse IgG (1:5000).

### qPCR

Neurospheres RNA was extracted with E. Z.N.A Total RNA I kit (Omega bio-tek), and cDNA was synthesized from 1 mg of RNA using the iScript Reverse Transcriptase Kit (Bio-Rad) according to the manufacturer’s protocols. Quantitative PCR was performed using SsoAdvanced universal SYBR green supermix (Bio-Rad). *Rplp0* RNA was used as an endogenous control for all reactions, and all reactions were performed in triplicate. Quantitative PCR was performed and analyzed using the CFX96 Touch Real-Time PCR Detection System (Bio-Rad). The primers used were the following: Nfia forward: 5′-TTG​GAC​CTC​GTC​ATG​GTG​ATC-3′, Nfia reverse: 5′-TGG​ACA​CAG​AGC​CCT​GGA​TTA-3′, Sox9 forward: 5′-CCA​ACA​TTG​AGA​CCT​TCG​ACG​T-3′, Sox9 reverse: 5′-ATG​CCG​TAA​CTG​CCA​GTG​TAG​G-3′, Zbtb20 forward: 5′-AAC​GCA​ATG​AAT​CCG​AGG​AGT-3′, Zbtb20 reverse: 5′-CCC​AAA​CTG​TTG​CTC​CAC​TGA-3′, Glt1 forward: 5′-TGA​ATG​AAA​CCA​TGA​ACG​AGG​C-3′, Glt1 reverse: 5′-GCC​GAA​AGC​AAT​AAA​GAA​TCC​GA-3′, Aldh1L1 forward: 5′-AGC​CAC​CTA​TGA​GGG​CAT​TC-3′, Aldh1L1 reverse: 5′-TGA​GTG​TCG​AGT​TGA​AAA​ACG​TC-3′, Sox10 forward: 5′-TCT​CAC​GAC​CCC​AGT​TTG​ACT-3′, Sox10 reverse: 5′-GCC​CCA​TGT​AAG​AAA​AGG​CTG-3′, Olig1 forward: 5′-GCA​GCC​ACC​TAT​CTC​CTC​ATC-3′, Olig1 reverse: 5′-CGA​GTA​GGG​TAG​GAT​AAC​TTC​GC-3′, Olig2 forward: 5′-GGG​AGG​TCA​TGC​CTT​ACG​C-3′, Olig2 reverse: 5′-CTC​CAG​CGA​GTT​GGT​GAG​C-3′, RPLP0 forward: 5′-CTG​CTG​AAC​ATG​CTG​AAC​ATC-3′, and RPLP0 reverse: 5′-GTC​GAG​CAC​TTC​AGG​GTT​AT-3’.

### Statistical analysis

All data were collected from at least four independent experiments. Results are presented as the mean ± SEM. Two group comparisons were performed using the Mann-Whitney test, and one-way ANOVA evaluated multiple groups following Tukey’s procedure. Differences were defined as statistically significant when *p* < 0.05.

## Results

### Conditional ablation of PTPRD increases embryonic neurogenesis

**FIGURE 1:**
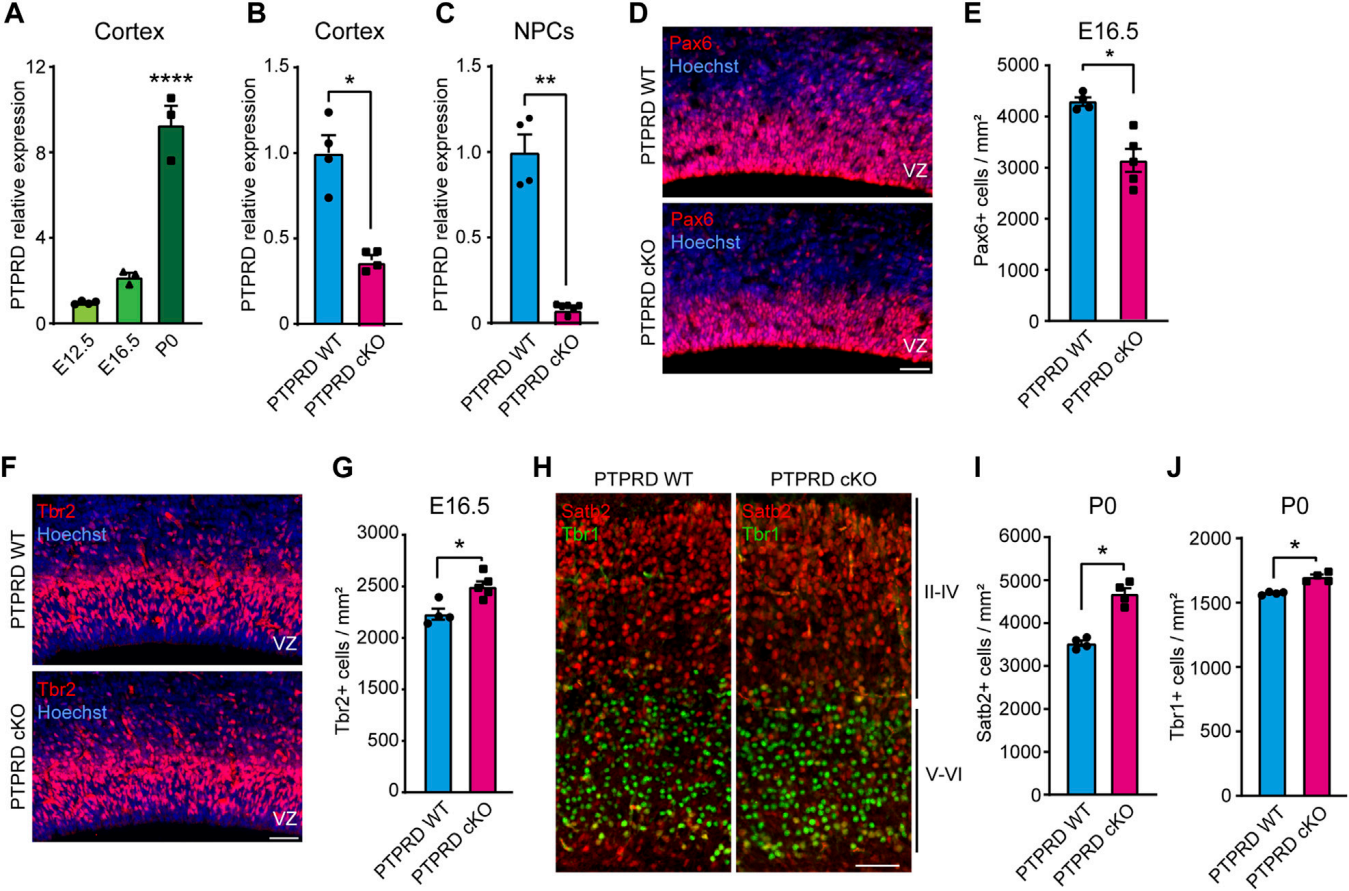
Embryonic neurogenesis is increased in the cortex of PTPRD cKO mice. **(A–C)** *Ptprd* relative expression was measured by qPCR in the brain cortex of WT mice at E12.5, E16.5, and P0 **(A)**, at E16.5 PTPRD WT and PTPRD cKO brain cortices **(B)**, and at E16.5 PTPRD WT and PTPRD cKO 7 DIV neurosphere cultures **(C)**. *p < 0.05; **p < 0.01; ****p < 0.0001; n = 3–4. **(D, E)** Pax6+ cells (red) were immunolabeled in the brain cortex of PTPRD WT and cKO mice at E16.5, where sections were counterstained with Hoechst 33,258 in blue **(D)** and quantified **(E)**. *p < 0.05; n = 4–5. **(F, G)** Tbr2+ cells (red) were immunolabeled in the brain cortex of PTPRD WT and cKO mice at E16.5, where sections were counterstained with Hoechst 33,258 in blue **(F)** and quantified **(G)**. *p < 0.05; n = 4–5. **(H–J)** Satb2+ (red) and Tbr2+ (green) cells were immunolabeled in the somatosensory cortex of PTPRD WT and cKO mice at P0 **(H)** and then quantified **(I, J)**. *p < 0.05; n = 4. Scale bar for **(D, F, H)** = 50 µm. In all panels, the error bars denote SEMs.

We have previously demonstrated that PTPRD regulates embryonic neurogenesis in the mouse brain (Tomita et al., 2020). Since most cortical glial cells emerge from the same pool of NPCs (Zhang et al., 2020; Li et al., 2021; Lin et al., 2021; Shen et al., 2021), we asked whether PTPRD was also involved in cortical gliogenesis. First, we analyzed *Ptprd* mRNA expression in embryonic and postnatal mouse cortex. qPCR results showed that *Ptprd* is increasingly expressed from E12.5 to P0 (Figure 1A) and that its expression is significantly upregulated at P0 when neurogenesis is almost completed, and gliogenesis reaches its peak, which is consistent with previous observations (Tomita et al., 2020; Blake et al., 2021).

To determine the role of PTPRD in cortical NPCs biology, we used a PTPRD cKO mouse that lacks *Ptprd* expression in the developing forebrain. When we characterized *Ptprd* expression in this novel mouse model, we observed that transcripts were reduced by more than half at E16.5 (Figure 1B) when gliogenesis started. At this embryonic stage, some cells derived from dorsal structures are expected to have migrated to the forebrain (Gorski et al., 2002; Clavreul et al., 2019; Shen et al., 2021; Clavreul et al., 2022), which explains why *Ptprd* expression is not fully abolished in the PTPRD cKO mouse cortex. Moreover, we cultured E16.5 cortical NPCs as neurospheres and measured *Ptprd* expression after 7 DIV (Figure 1C), finding that forebrain NPCs derived from PTPRD cKO embryos lacks *Ptprd* expression.

Then, to address whether the conditional ablation of *Ptprd* expression in forebrain NPCs phenocopies our previous observations, aberrantly increasing cortical neurogenesis (Tomita et al., 2020), we analyzed neurogenic progenitors and neurons in the PTPRD cKO mouse cortex. We quantified radial glial precursors immunolabeled with Pax6 (Figure 1D), observing a reduced number of Pax6+ cells in the PTPRD cKO cortex at E16.5 (Figure 1E). Also, we quantified the neuronal intermediate progenitor cells immunolabeled with Tbr2 (Figure 1F), where an increase was observed in the PTPRD cKO cortex at E16.5 (Figure 1G). We also immunolabeled neuronal populations positive for Satb2 and Tbr1 in the somatosensory cortex at P0 (Figure 1H), observing a significant increase in both populations for PTPRD cKO mice (Figures 1I, J), as previously described (Tomita et al., 2020). Our results confirm that conditional ablation of *Ptprd* expression in the mouse forebrain also exacerbates cortical neurogenesis in our mouse model. Besides, we observed a reduced number of radial glial cells in the cortex of PTPRD cKO embryos at E16.5, suggesting a reduced NPCs pool available at the onset of gliogenesis.

### Loss of *Ptprd* expression in the forebrain impairs cortical gliogenesis *in vivo*

**FIGURE 2:**
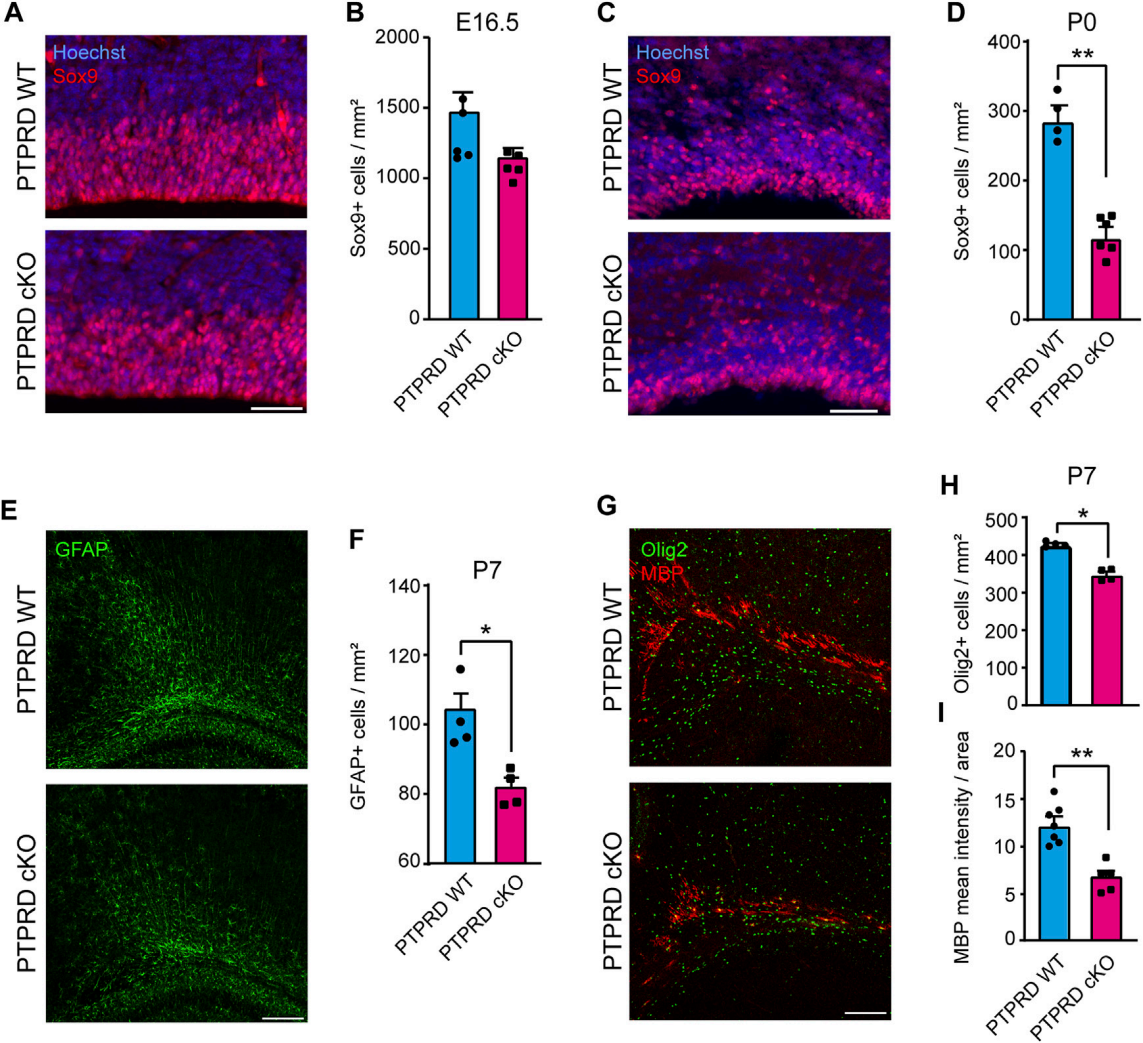
Conditional ablation of PTPRD reduces the number of gliogenic precursor and glial cells *in vivo*. **(A–D)** Gliogenic precursors were immunolabeled with Sox9 (red), and sections were counterstained with Hoechst 33,258 (blue) at E16.5 **(A)** and P0 **(C)**. The number of Sox9+ cells was quantified at each stage **(B, D)**. **p < 0.01; n = 4–5. **(E, F)** Astrocytes were immunolabeled with GFAP (green) at P7 **(E)**, and the number of GFAP + cells in the motor/somatosensory cortex was quantified **(F)**. *p < 0.05; n = 4. **(G–I)** Oligodendrocytes were immunolabeled with Olig2 (green) and MBP (red) in the corpus callosum at P7 **(G)**, and the number of Olig2+ cells were quantified **(H)**, while MBP mean fluorescence was measured in the total area of the corpus callosum **(I)**. *p < 0.05; **p < 0.01; n = 4–5. Scale bar for **(A)** and **(C)** = 50 µm. Scale bar for **(E)** and **(G)** = 100 µm. In all panels, the error bars denote SEMs.

To determine whether conditional ablation of *Ptprd* expression impacts glial cell development, we quantified glial cell populations in the forebrain of PTPRD cKO mice. First, we analyzed the number of positive cells for the glial cell precursor marker Sox9 at different stages. At E16.5 (Figure 2A), we observed a mild, non-significant reduction of Sox9+ cells in the PTPRD cKO cortex (Figure 2B). However, the number of Sox9+ cells (Figure 2C) was significantly reduced in the PTPRD cKO cortex at P0 (Figure 2D), suggesting that PTPRD regulates cortical gliogenesis in mice.

Then, we assessed the number of differentiated glia in the postnatal cortex of PTPRD cKO mice. We quantified the number of astrocytes stained with GFAP at P7 in the motor/somatosensory cortex (Figure 2E) and found that PTPRD cKO mice had fewer astrocytes (Figure 2F). We also evaluated the number of oligodendrocytes and oligodendrocyte precursor cells (OPCs) that were stained with Olig2 (Figure 2G, green) and observed a significant reduction in oligodendroglia populations in the cortex of PTPRD cKO mice at this stage (Figure 2H). Furthermore, we analyzed myelinization in the corpus callosum at P7 (Figure 2G, red) by measuring myelin binding protein (MBP) immunolabeling intensity, normalized for the total area occupied by Olig2/MBP-positive cells. Interestingly, we found significantly reduced MBP intensity in the corpus callosum of PTPRD cKO mice (Figure 2I). Therefore, these data suggest that the lack of *Ptprd* expression in the forebrain reduces glial cell populations in the postnatal cortex.

### NPCs from PTPRD cKO mice show altered differentiation *in vitro*

**FIGURE 3:**
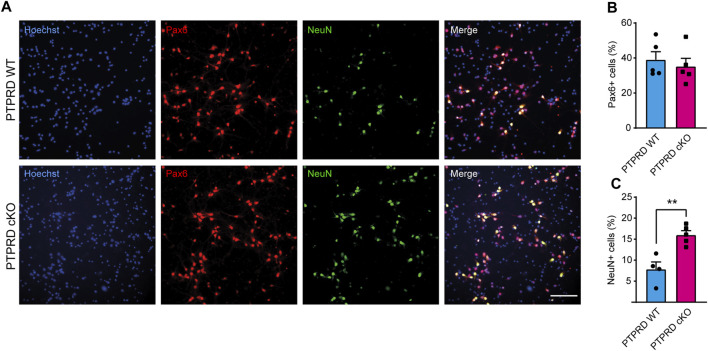
PTPRD cKO mice show increased neuronal differentiation *in vitro*. **(A–C)** Cortical NPCs obtained from E16.5 PTPRD WT and cKO embryos were grown as neurospheres and, after 7 DIV, were dissociated and plated as monolayers. 7 days later, cells were fixed and immunostained for Pax6 (red) and NeuN (green) and counterstained with Hoechst 33,258 in blue **(A)**. The percentage of Pax6+ **(B)** and NeuN+ cells **(C)** were quantified relative to the total cells in the culture, identified by Hoechst staining. **p < 0.01; n = 4–5. Scale bar for **(A)** = 100 µm. In all panels, the error bars denote SEMs.

**FIGURE 4:**
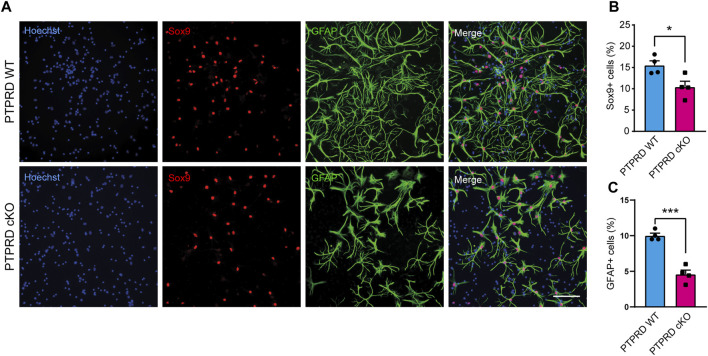
PTPRD cKO mice show impaired astrocytic differentiation *in vitro*. **(A–C)** Cortical NPCs obtained from E16.5 PTPRD WT and cKO embryos were grown as neurospheres and, after 7 DIV, were dissociated and plated as monolayers. 7 days later, cells were fixed and immunostained for Sox9 (red) and GFAP (green) and counterstained with Hoechst 33,258 in blue **(A)**. The percentage of Sox9+ **(B)** and GFAP+ cells **(C)** were quantified relative to the total cells in the culture, identified by Hoechst staining. *p < 0.05; ***p < 0.001; n = 4. Scale bar for **(A)** = 100 µm. In all panels, the error bars denote SEMs.

To understand how PTPRD regulates gliogenesis, we evaluated whether glial differentiation was also impaired in cortical NPCs lacking *Ptprd* expression in culture. To address this, we cultured E16.5 cortical NPCs as neurospheres for 7 DIV to maintain their stemness, and then, neurospheres were disaggregated and plated in conditions that allowed them to differentiate into glial precursors, astrocytes, oligodendrocytes, or neurons (Dittmann et al., 2022). After 7 days of differentiation (DOD), we analyzed the number of NPCs differentiated into neurons (Figure 3) and astrocytes (Figure 4). To assess this, we immunolabeled radial glial cells with Pax6 and neurons with NeuN (Figure 3A) to quantify the percentage of radial glia and differentiated neurons, respectively, finding that PTPRD cKO cultures have an increased percentage of neurons (Figure 3C) without changing the percentage of Pax6+ radial glial cells relative to the total of cells in the culture, identified by Hoechst staining (Figure 3B). However, if we quantify the proportion of Pax6+ cells over the sum of Pax6+ and NeuN+ cells, we found a significant reduction in PTPRD cKO compared to PTPRD WT cultures (n = 5; *t*-test *p* = 0.0317), which is consistent with our previous observations (Tomita et al., 2020).

**FIGURE 5:**
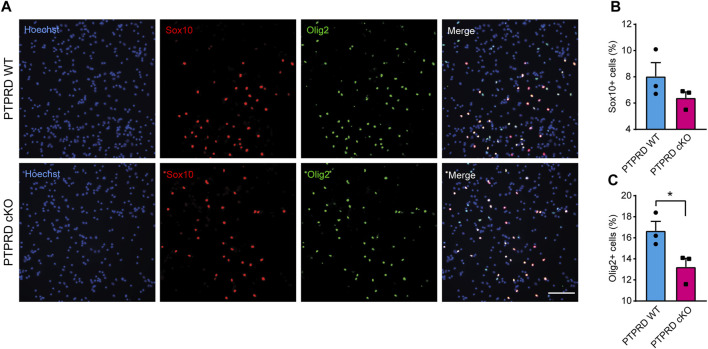
Conditional ablation of PTPRD impaired oligodendrocytic differentiation *in vitro*. **(A–C)** Cortical NPCs obtained from E16.5 PTPRD WT and cKO embryos were grown as neurospheres and, after 7 DIV, were dissociated and plated as monolayers. 14 days later, cells were fixed and immunostained for Sox10 (red) and Olig2 (green) and counterstained with Hoechst 33,258 in blue **(A)**. The percentage of Sox10+ **(B)** and Olig2+ cells **(C)** were quantified relative to the total cells in the culture, identified by Hoechst staining. *p < 0.05; n = 3. Scale bar for **(A)** = 100 µm. In all panels, the error bars denote SEMs.

Then, we also immunolabeled cells for the glial precursor marker Sox9 and the astrocytic marker GFAP (Figure 4) and observed that PTPRD cKO cultures had a reduced percentage of Sox9+ (Figure 4B) and GFAP+ cells (Figure 4C) after 7 DOD. Moreover, we also allowed plated NPCs to differentiate for 14 days and labeled the cells for Sox10 and Olig2 (Figure 5A) to assess the percentage of cells differentiated into OPCs and oligodendrocytes, respectively. We found that PTPRD cKO cultures had a reduced percentage of Olig2+ cells (Figure 5C) after 14 DOD and a mild reduction in Sox10+ cells (Figure 5B). Together, these results suggest that cortical NPCs lacking *Ptprd* expression differentiates predominantly into neurons rather than glia.

### Conditional ablation of PTPRD impairs the gliogenic potential of NPCs

**FIGURE 6:**
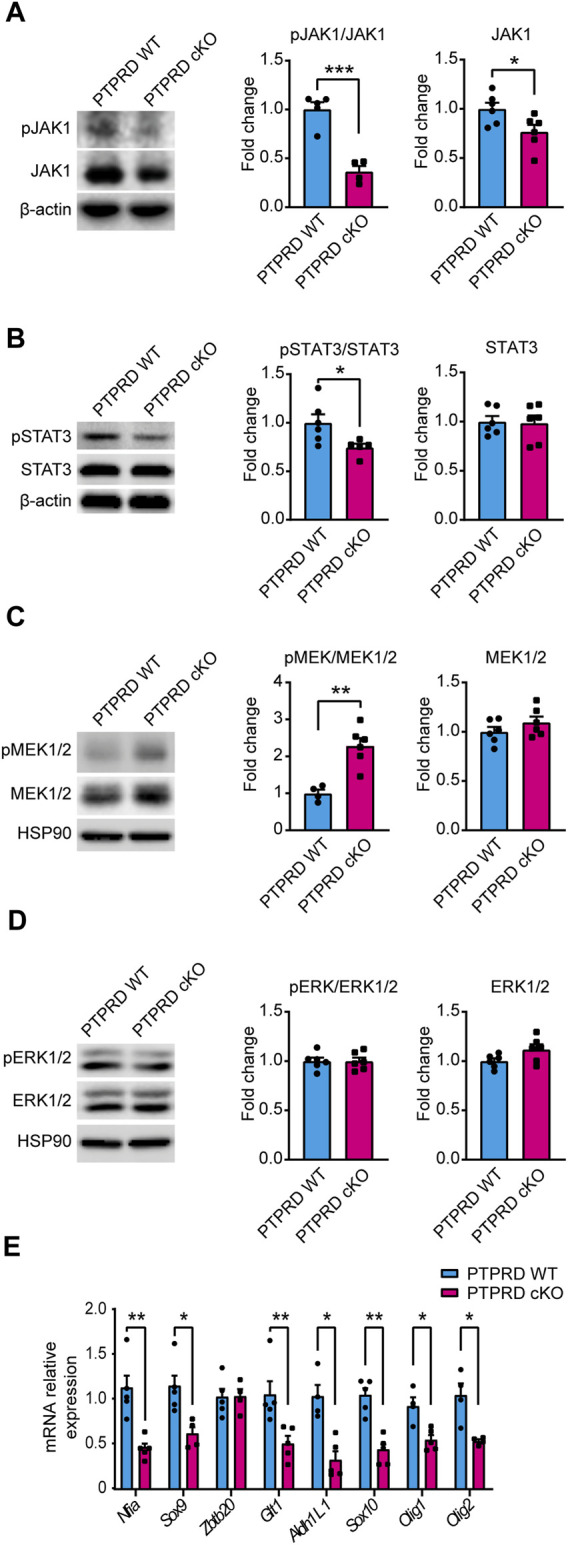
Neural precursor cells lacking PTPRD expression have impaired gliogenic signaling. **(A–E)** Cortical NPCs obtained from E16.5 PTPRD WT and cKO embryos were grown as neurospheres and, after 7 DIV, were lysed for Western blot **(A–D)** or qPCR **(E)** assays. **(A, B)** Gliogenic signaling pathway activation was assessed by Western blot for pJAK1 **(A)** and pSTAT3 **(B)**. Representative immunoblots for pJAK1, total JAK1, and β-actin (loading control), and the relative quantification of band intensity are shown in **(A)**. Representatives immunoblot for pSTAT3, total STAT3, and β-actin (loading control), and the relative quantification of band intensity is shown in **(B)**. **(C, D)** Neurogenic signaling pathway activation was assessed by Western blot for pMEK1/2 **(C)** and pERK1/2 **(D)**. Representative immunoblots for pMEK1/2, total MEK1/2, and HSP90 (loading control) and the relative quantification of band intensity are shown in **(C)**. Representative immunoblots for pERK1/2, total ERK1/2, and HSP90 (loading control) and the relative quantification of band intensity are shown in **(D)**. In panels **(A–D),** relative quantifications of band intensity are represented as fold change of the control group (PTPRD WT). **(E)** mRNA relative expression of the gliogenic genes *Nfia, Sox9, Zbtb20, Glt1, Aldh1L1, Sox10, Olig1* and *Olig2* were assessed by qPCR. *p < 0.05; **p < 0.01; ***p < 0.001; n = 4–6. In all panels, the error bars denote SEMs.

To determine the molecular mechanism by which PTPRD cKO NPCs have impaired gliogenic differentiation, we analyzed the main signaling pathways that drive gliogenesis and neurogenesis in the CNS by Western blot from 7 DIV neurosphere cultures lysates (Figure 6). The JAK/STAT gliogenic signaling pathway was evaluated by measuring the protein levels and activation of JAK1 (Figure 6A) and STAT3 (Figure 6B) and observed a reduction in the phosphorylation of both proteins, with a reduced expression of JAK1 in PTPRD cKO neurospheres. Then, we also examined the neurogenic signaling pathway MEK/ERK. We found that PTPRD cKO neurospheres had an increased phosphorylation of MEK1/2 (Figure 6C), as previously described (Tomita et al., 2020), with no changes in ERK1/2 (Figure 6D). Furthermore, we quantified the relative expression of relevant glial genes from 7 DIV neurosphere cultures and observed that the expression of *Nfia*, *Sox9*, *Glt1*, *Aldh1L1*, *Sox10*, *Olig1*, *Olig2* was downregulated in PTPRD cKO neurospheres (Figure 6E). Therefore, these data suggest that cortical PTPRD cKO NPCs has a decreased gliogenic potential due to increased and persistent neurogenic signaling.

## Discussion

The development of the cerebral cortex involves a series of highly regulated cellular and molecular mechanisms that give rise to the different populations of cells that compose it. Our research aimed to study the role of PTPRD in cortical gliogenesis, shedding light on the impact of *PTPRD* mutations in the development of glial cells. Based on our previous work demonstrating the critical role of PTPRD in cortical neuron development (Tomita et al., 2020), we extended our analysis to assess its involvement in gliogenesis, given the evidence suggesting common origins of cortical neurons and glial cells (Zhang et al., 2020; Li et al., 2021; Lin et al., 2021; Shen et al., 2021). Using a PTPRD cKO model, we confirmed that the absence of PTPRD in telencephalic NPCs increases cortical neurogenesis, evidenced by increased intermediate progenitors and mature neurons, and reduces radial glial cells in the cortex at E16.5 (Figure 1). Also, PTPRD cKO showed reduced glial populations in the embryonic and postnatal telencephalon (Figure 2), which could be explained by the reduced pool of remaining NPCs available at the beginning of gliogenesis and by a predominant neuronal differentiation in cortical NPCs that lacks *Ptprd* expression, as our *in vitro* differentiation assays results suggest (Figures 3–5). In the search for molecular mechanisms that could be inducing these glial impairments, we found a reduced activation in the gliogenic signaling JAK/STAT (Bonni et al., 1997; He et al., 2005; Hong and Song, 2014; Sloan and Barres, 2014; Han-Chung et al., 2016; Lee et al., 2016; Takouda et al., 2017), as well as an increased activation of the neurogenic signaling mediator MEK1/2 (Ménard et al., 2002; Paquin et al., 2005; Namihira and Nakashima, 2013; Vithayathil et al., 2018; Tomita et al., 2020), which was accompanied by a downregulation of several glial genes in PTPRD KO cortical NPCs (Figure 6).

We have previously demonstrated that constitutive PTPRD KO mice had aberrant cortical neurogenesis (Tomita et al., 2020). Here, we developed a PTPRD cKO mice that lack its expression only in NPCs that originate brain cortex to address if part of the observed phenotype in the constitutive PTPRD KO mice could be due to the absence of PTPRD in other brain regions or tissues, especially given that from E16.5 onwards, cells that are not originated in the cortex have migrated into this structure (Gorski et al., 2002; Swinnen et al., 2013; Shen et al., 2021). By eliminating PTPRD expression only in EMX1+ cells, the specific role of PTPRD in cortical neurodevelopment can be studied, providing greater precision in the interpretation of results and allowing to address the role of PTPRD in cortical NPCs with higher specificity and in a cell-autonomous fashion, regardless of the influence that could exert the cells that invade and interact with the cortex during its development.

Importantly, our current PTPRD cKO mouse model also showed increased numbers of intermediate progenitors and neurons in the mouse brain cortex (Figure 1), confirming that cortical NPCs lacking *Ptprd* expression has increased neuronal differentiation. We also found that loss of PTPRD expression in telencephalic NPCs impairs the number of cortical radial glia (Pax6+ cells, Figure 1) at E16.5, which is consistent with our previous observations (Tomita et al., 2020), suggesting that PTPRD would have a role in radial glial cell maintenance at gliogenesis onset. The molecular mechanism by which PTPRD variants would promote aberrant cortical neurogenesis is through the aberrant activation of the receptor tyrosine kinases PDGFRβ and TrkB and their intracellular mediators MEK/ERK during neurogenesis (Tomita et al., 2020). Although analyzing all the cellular and molecular mechanisms by which PTPRD regulates neurogenesis is beyond the objectives of this study, our current and previous results suggest that PTPRD would mediate this process in a cell-intrinsic manner. Interestingly, neurodevelopmental disorders related to increased cortical neurons are often associated with impaired neuronal phenotypes and behavioral alterations (Fang et al., 2014). Regarding the role of PTPRD in neurogenesis, our observations align with this hypothesis since previous studies have found that PTPRD KO mice have aberrant dendritic processes (Nakamura et al., 2017), impaired synaptic development and function (Takahashi et al., 2012; Park et al., 2020; Sclip and Südhof, 2020), and behavioral deficits (Drgonova et al., 2015; Uhl et al., 2018; Park et al., 2020; Ho et al., 2023), which could explain the contribution of PTRPD mutations to neurodevelopmental disorders (Schormair et al., 2008; Elia et al., 2010; Pinto et al., 2010; Levy et al., 2011; Yang et al., 2011; Gai et al., 2012; Gazzellone et al., 2016; Liu et al., 2016; Li et al., 2018; Li et al., 2023; Lyulcheva-Bennett and Bennett, 2023).

Given the evidence showing that NPCs would have the same potential to generate neurons and glial cells and that this differentiation would depend solely on stochasticity and epigenetic changes induced by the developmental stage (Miller and Gauthier, 2007a; Zhang et al., 2020; Li et al., 2021; Lin et al., 2021; Shen et al., 2021), we wondered whether PTPRD cKO cortical NPCs that remain after neurogenesis would have alterations in glial development. Unlike what was observed in neurogenesis, the absence of PTPRD in the telencephalon had a detrimental impact on cortical gliogenesis. Reductions in Sox9+ glial precursor cells were observed at E16.5, which becomes significant at P0 when gliogenesis peaks (Figure 2). Additional results showed decreased astrocytes and oligodendrocytes in the PTPRD cKO postnatal cortex, which correlates with reduced myelin expression in the corpus callosum. Since most perinatal cortical astrocytes and their precursors originate from cortical NPCs and astrocyte local proliferation (Tsai et al., 2012; Clavreul et al., 2019; Vogel and Wegner, 2021; Clavreul et al., 2022), the reduced number of radial glia cells (Pax6+) and astrocytic progenitors (Sox9+) (Kang et al., 2012; Martini et al., 2013; Sloan and Barres, 2014), and the postnatal reduction of GFAP+ astrocytes observed in PTPRD cKO mouse cortex indicates that PTPRD has a role in astrogliogenesis. This observation can also be extended to oligodendrogenesis since most OPCs and oligodendrocytes originated from EMX1+ NPCs are in the main areas studied here, such as deep cortical layers and white matter (Shen et al., 2021), suggesting that PTPRD would also regulate oligodendrogenesis. However, previous studies have shown that PTPRD ablation does not significantly affect the differentiation of oligodendrocytes in the spinal cord, affecting myelination only (Zhu et al., 2015), which suggests that different molecular mechanisms mediate cortical oligodendrogenesis in the CNS.

This impaired developmental gliogenesis could have profound implications in the adult brain since after P7, cortical astrocytes do not proliferate locally (Clavreul et al., 2019), leading to several disruptions in brain function, such as neurotransmitter imbalances, impaired synaptic function, disrupted brain circuits, and worsen neuronal physiology (Clavreul et al., 2022). As for oligodendrocytes, since OPCs are the most proliferative cells in the brain and maintain their stemness until adulthood (Goldman and Kuypers, 2015), further research is needed to determine if the absence of PTPRD in cortical NPCs produces a reduced oligodendrogenesis that is sustained over time, or if it only delays it as has been seen in the spinal cord (Zhu et al., 2015). PTPRD cKO mice showed a reduced proportion of cortical Pax6+ cells remaining after neurogenesis (Figure 1), explaining the reduced number of glia in the postnatal cortex. Besides, we wondered if these remaining radial glial cells could also have an impaired gliogenic differentiation potential. To address this, we conducted *in vitro* differentiation assays using cortical NPCs from PTPRD cKO mice (Figures 3–5). Cultured cortical NPCs that lack PTPRD expression exhibited a differentiation bias toward neurons, disfavoring glial lineages. This was evidenced by an increase in the proportion of NPCs differentiated to neurons (NeuN + cells, Figure 3) and a reduction in the proportion of glial and glial progenitor cells (Figures 4, 5). This confirms that the cortical NPCs from PTPRD cKO mice remaining after neurogenesis have impaired glial differentiation potential, corroborating the importance of PTPRD in cortical gliogenesis. Since our experimental strategy does not include pro-gliogenic or neurogenic ligands in the cell culture media that could promote a directionality in differentiation (Dittmann et al., 2022), these results suggest that cortical NPCs have a cell-autonomous differentiation program. However, it cannot be ruled out that the diverse cell populations present in the culture could be influencing the differentiation of remaining NPCs, an effect that would possibly be mediated by PTPRD ligand properties as observed in synaptic differentiation (reviewed in Cornejo et al., 2021), and that should be assessed in future studies.

Further exploration of the molecular underpinnings of gliogenesis revealed that cortical NPCs from PTPRD cKO mice have decreased gliogenic signaling, as demonstrated by the downregulation of key glial genes and the reduction in JAK/STAT signaling activation (Figure 6), which is crucial for gliogenesis (Bonni et al., 1997; He et al., 2005; Hong and Song, 2014; Sloan and Barres, 2014; Han-Chung et al., 2016; Lee et al., 2016; Takouda et al., 2017). Evidence shows that one of the major substrates for PTPRD phosphatase activity is STAT3 (Ortiz et al., 2014; Kim et al., 2018). Therefore, we expected to observe an increased STAT3 phosphorylation in PTPRD cKO mice. Interestingly, cortical NPCs lacking *Ptprd* expression shows reduced activation of STAT3, suggesting that PTPRD participation in JAK/STAT signaling could be by modulating JAK1 expression and activation (Figure 6), which would explain the reduced activation of JAK1-downstream mediator STAT3 (He et al., 2005; Hu et al., 2021).

We also observed that gliogenic impairments in our PTPRD cKO model are associated with an increased activation of MEK1/2 in cortical NPCs. However, we did not find changes in ERK1/2 activation, unlike what was previously observed in constitutive PTPRD KO NPCs at E14 (Tomita et al., 2020). The interpretation of these results is puzzling since impaired gliogenesis has been observed in NPCs deficient in MEK1/2 at E16 (Li et al., 2012), which would imply that an increase in MEK1/2 activation would lead to a rise in gliogenesis. However, evidence shows that MEK activation is crucial for neurogenesis at earlier embryonic stages (Ménard et al., 2002; Tomita et al., 2020), which correlates with the increased neurogenesis observed in our model. This allows us to speculate that at E16.5, cortical NPCs lacking PTPRD expression still has some neurogenic signaling activated. Thus, when endogenous ERK activation has reached its maximum (Paquin et al., 2005), MEK signaling promotes extended neurogenesis. However, further work is needed to assess the temporality in the activation of neurogenic and gliogenic signaling pathways in our PTPRD cKO model to address a potential delay in the onset of gliogenesis induced by loss of *Ptprd* expression.

These data suggest that PTPRD would participate in intracellular signaling, mediating neuro and gliogenesis. The mechanism by which PTPRD mediates cortical neurogenesis was partially elucidated previously by Tomita et al., 2020, where we demonstrated that PTPRD directly regulates the catalytic activity of two important kinases regulating NPCs proliferation and differentiation: PDGRFβ and TrkB (Bartkowska et al., 2007; Xu et al., 2013). PTPRD ablation increases PDGRFβ and TrkB activation, which upregulates MEK/ERK activation (Stephens et al., 1994; Rönnstrand et al., 1999) and exacerbates neurogenesis (Tomita et al., 2020). Since *Ptprd* mRNA expression increases during cortical development, this neurogenic signaling pathway mediated by PTPRD could be regulated epigenetically across embryonic development. However, an effect mediated by embryonic extracellular ligands interacting with PTPRD cannot be ruled out, given its receptor properties (Cornejo et al., 2021). As for gliogenesis, the mechanism by which PTPRD would be participating is less clear since its interaction with gliogenic signaling mediators other than STAT3 (Veeriah et al., 2009; Ortiz et al., 2014; Peyser et al., 2015; Kim et al., 2018), has not been proven as for our knowledge. Moreover, since PTPRD ablation reduces the activation of JAK1 and STAT3 in our model, we predict that an intermediary regulatory protein would mediate this effect to produce this downregulation in JAK/STAT signaling. Unfortunately, we could not observe changes in the activation of upstream gliogenic mediators such as gp130 and LIFRβ (Miller and Gauthier, 2007) (data not shown). Therefore, further research is needed to fully determine the molecular mechanism by which PTPRD would participate in regulating cortical gliogenesis.

### Concluding remarks

These findings collectively suggest that PTPRD deficiency in cortical NPCs prevents the correct development of the glial cell populations during brain development and proposes a link between PTPRD and the signaling pathways that govern gliogenesis, highlighting the role of PTPRD in keeping the balance in the generation of neurons and glial cells in the developing brain cortex. Importantly, dysregulation of this balance could be implicated in several neurodevelopmental disorders, including Noonan syndrome (Gauthier et al., 2007), Neurofibromatosis-1 (Hegedus et al., 2007), Costello syndrome (Paquin et al., 2009), Cardiofaciocutaneous syndrome (Urosevic et al., 2011), and Down syndrome (Zdaniuk et al., 2011). Also, it is important to highlight that alterations in glia also impair neuronal function, such has been observed in Rett syndrome (Ballas et al., 2009; Lioy et al., 2011), Fragile-X syndrome (Jacobs et al., 2010), schizophrenia (Gittins and Harrison, 2011), and other neuropsychiatric disorders (reviewed in Milbocker et al., 2021; Rahman et al., 2022), emphasizing the importance of studying the contribution of possible alterations in gliogenesis to understand the etiology of brain disorders.

Since PTPRD mutations have been associated with several brain pathologies (Schormair et al., 2008; Pinto et al., 2010; Elia et al., 2010; Levy et al., 2011; Yang et al., 2011; Gai et al., 2012; Gazzellone et al., 2016; Liu et al., 2016; Li et al., 2018; Cornejo et al., 2021; Li et al., 2023), it is important to consider that part of the behavioral alterations observed in them could be due to the deregulation of the neuron/glia balance and to address potential therapeutic targets that allow reestablishing this cellular equilibrium. Molecular impairments observed in NPCs that lack PTPRD expression highlight potential therapeutic targets for disorders in which impaired glial development contributes to their pathogenesis. However, further research is needed to assess experimental strategies that allow rescuing the cortical phenotype induced by PTPRD mutations to address the detailed molecular mechanisms through which PTPRD modulates neural differentiation. In summary, our research provides knowledge regarding the multi-faceted role of PTPRD in neurodevelopment and shows that the interaction between PTPRD and the signaling pathways that govern this process are important targets for future research and therapeutic interventions.

## Data Availability

The raw data supporting the conclusion of this article will be made available by the authors, without undue reservation.
